# Diagnosis and Treatment of Chronic Non-bacterial Osteomyelitis: A Survey on Current Practices Adopted by Pediatric Rheumatologists in Saudi Arabia

**DOI:** 10.7759/cureus.37103

**Published:** 2023-04-04

**Authors:** Jubran Alqanatish, Lina A Bayazeed, Lujain Alahmadi, Mesaed AlSenani, Omar Aldibasi

**Affiliations:** 1 Pediatric Rheumatology, King Abdullah Specialist Children’s Hospital, Riyadh, SAU; 2 Pediatric Rheumatology, King Saud Bin Abdulaziz University for Health Sciences, Riyadh, SAU; 3 Department of Biostatistics and Bioinformatics, King Abdullah International Medical Research Center, Riyadh, SAU

**Keywords:** treatment, survey, saudi arabia, diagnosis, cno, chronic nonbacterial osteomyelitis

## Abstract

Introduction: Chronic nonbacterial osteomyelitis (CNO) is an autoinflammatory bone disease usually managed by pediatric rheumatologists (PRs). There is a need for a consensus treatment plan to minimize the diversity in clinical practice in the diagnosis and management of CNO. In this study, we explored the practice of PRs in Saudi Arabia on the diagnosis and treatment of patients with CNO.

Methods: This is a cross-sectional study that was conducted among PRs in Saudi Arabia (May to September 2020). A survey was performed among PRs registered in the Saudi Commission for Health Specialties using an electronic-based questionnaire. The survey consisted of 35 closed-ended questions about the diagnosis and management of CNO patients. We explored the approaches adopted by PRs in diagnosing and monitoring the disease activity, their awareness of clinical possibilities that necessitate ordering bone biopsy, and the treatment choices they considered for CNO patients.

Results: We scrutinized data from a total of 77% (n=41/53) PRs who responded to our survey. Magnetic resonance imaging (MRI) was reported as the most frequently used modality in suspected CNO (82%, n=27/33), followed by plain X-ray (61%) and bone scintigraphy (58%). Magnetic resonance imaging of a symptomatic site is the imaging modality of choice for the diagnosis of CNO (82%), Followed by X-ray (61%) and bone scintigraphy (58%). The reasons for performing bone biopsy were unifocal lesions (82%), unusual sites of presentation (79%), and multifocal lesions (30%). The preferred treatment regimens were bisphosphonates (53%), non-steroidal anti-inflammatory drugs alone (43%), or biologics with bisphosphonates (28%). The reasons to upgrade the treatment in CNO included the development of vertebral lesions (91%), the development of new lesions in MRI (73%), and the elevation of inflammatory markers (55%). The disease activity was assessed by history and physical examination (91%), inflammatory markers (84%), MRI of targeted symptomatic site (66%), and a whole-body MRI (41%).

Conclusions: The approach to diagnosis and treatment of CNO varies among PRs in Saudi Arabia. Our findings provide a background for the development of a consensus treatment plan for challenging CNO patients.

## Introduction

Chronic nonbacterial osteomyelitis (CNO) is a bone disease of unknown cause. In the CNO animal model, there is a clear defect in innate immunity, which makes it plausible to be classified as an autoinflammatory disorder [1,2]. The clinical manifestations of CNO include persistent bone pain, fever, and joint pain, and bony tenderness with or without local swelling in physical examination [3-5]. Radiological findings of mixed lytic and sclerotic bone lesions are suggestive but not pathognomonic of CNO [6-9]. In the absence of a consensus treatment plan, pediatric rheumatologists (PRs) face challenges in the management of CNO patients that lead to a wide variation in diagnostic and treatment approaches. For instance, the practice of requesting a bone biopsy varies and is usually considered whenever there is a concern about infection or malignancy [10-12]. Also, there are variations in practice amongst PRs regarding the choice of imaging modality to be ordered for CNO patients; such as a bone scan or MRI [6-9].

Another challenge for CNO management is the scarcity of trained PRs in Saudi Arabia. Pediatric Rheumatology service is unavailable in the peripheral cities in Saudi Arabia due to the clustering of PRs in the large centers in the main cities such as Riyadh, Jeddah, and Dammam. Currently, there are 41 attending PRs registered with the Saudi Commission for Health Specialties (SCFHS). The limited opportunity for a structured postgraduate training in pediatric rheumatology would certainly affect the development of a consensus treatment plan for rare clinical encounters such as CNO. Nevertheless, opportunities to develop such treatment plans among PRs in Saudi Arabia are available through their annual meeting at the Saudi Society of Rheumatology conferences, and two clinical forums that rotate annually in different cities. 

To our knowledge, this survey is the first to explore the practice of PRs in Saudi Arabia regarding an overall rare, yet important disease, that is not uncommonly seen in rheumatology clinics. We explored the approaches adopted by PRs in diagnosing and monitoring the disease activity, their awareness of clinical possibilities that necessitate ordering bone biopsy, and the treatment choices they considered for CNO patients.

## Materials and methods

We surveyed PRs who practice in nine out of 13 administrative regions in Saudi Arabia. An online survey was distributed using google forms among PRs registered in the SCFHS. The survey link was sent to the 41 attending PRs and 12 fellows using direct communication between May to September 2020. An introductory message and two subsequent follow-up messages over two weeks were sent over to participants. There were no financial incentives for participating in the study. We only included PRs who had reasonable experience in diagnosing and managing CNO, hence; we excluded the fellows (n=8, 19.5%) in the first year of training from the list of respondents.

The survey consisted of 35 closed-ended questions rated on a five-point Likert scale. The survey started with questions that verified the level of confidence and experience in dealing with CNO patients that were used as inclusion criteria, such as: "How frequently do you diagnose CNO? cases/year" and "How confident do you feel in making diagnosis of CNO?". Other items explored the approaches in diagnosing and monitoring the disease activity, identifying other diseases that can be a differential diagnosis and necessitate ordering a bone biopsy. Exploring the treatment choices in CNO in common practices by PRs in Saudi Arabia (survey was available at: https://tinyurl.com/3bb8sn6d). We designed this survey in order to achieve the above objectives. It was validated through face validity and content validity. The validity of the survey was assessed by a team of PRs lead by a senior consultant. The survey items were analyzed using descriptive statistics, including frequencies and percentages for categorical variables and means and standard deviations for continuous variables.

An ethical approval was obtained for this study from the Institutional Review Board (IRB) of King Abdullah International Medical Research Center (KAIMRC) (approval reference number: RC20/231/R, date of the approval: 13th May 2020).

## Results

**Figure 1:**
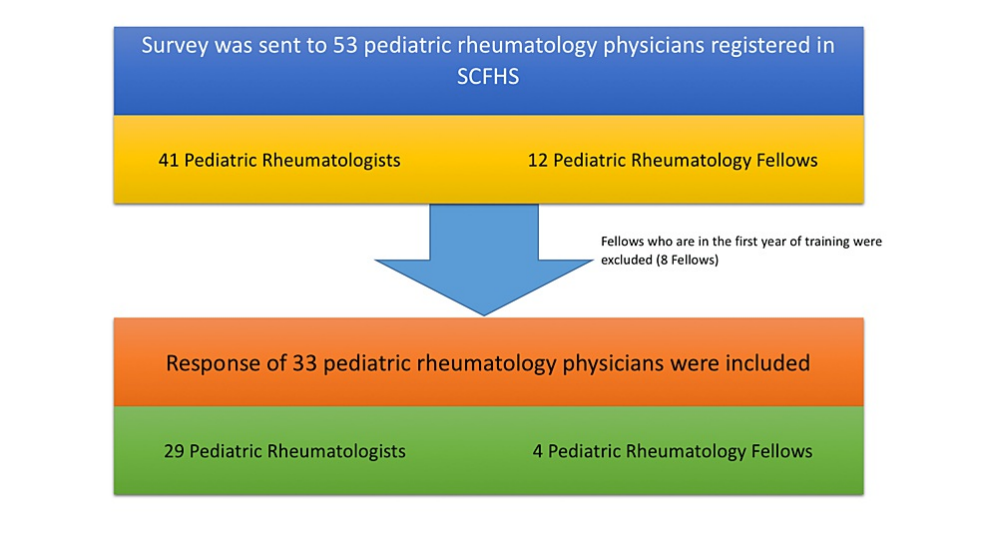
A representative scheme of the pediatric rheumatology physicians participating in the study SCFHS- Saudi Commission for Health Specialties

A total of 41/53 PRs (response rate of 77%) registered in the SCFHS answered the survey (Figure1). The participating physicians had a mean duration of practice of eight years (range, 0−27). Overall, 76% (n=25) of respondents felt “often” confident in diagnosing CNO (Table 1). CNO-associated conditions, such as inflammatory bowel disease, psoriasis, or uveitis were reported to be “mostly”, “never” or “rarely” associated with CNO, 58% (n=19), 45% (n=15), and 78% (n=26) respectively.

**Table 1:** Demographic data of the participants

Demographic characteristics	Category	N (%)
Gender	Male	15 (45.5)
	Female	18 (54.5)
Years of Experience	< 5 years	10 (30)
	>or= 5years	23 (70)
Number of cases seen/year	1-2 cases/year	25 (76)
	2-5 cases/year	8 (24)
Residency of the participants in Saudi Arabia	Central region	17 (52)
	Western region	8 (24)
	Other regions	8 (24)
Total		33 (100%)

**Figure 2:**
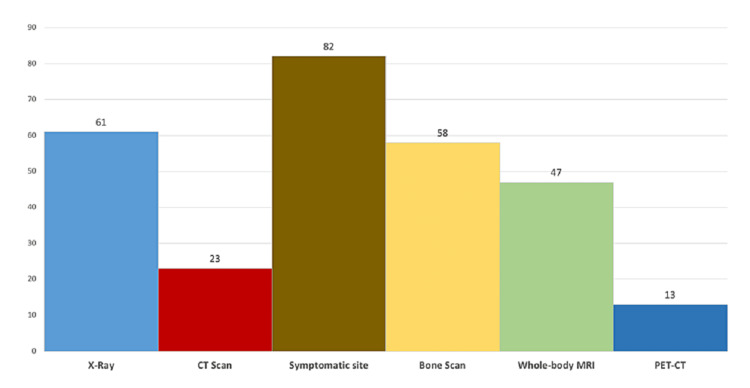
Imaging modality choices for diagnosing CNO CNO: Chronic nonbacterial osteomyelitis

**Figure 3:**
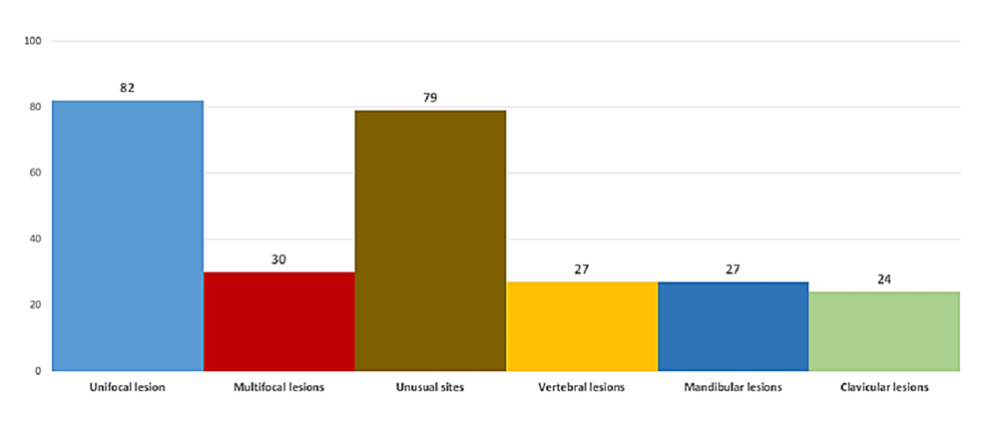
Indications for bone biopsy in CNO.

Among all imaging modalities used for ‘suspecting” CNO, magnetic resonance imaging (MRI) was the most commonly used (82%, n=27), followed by plain X-ray (61%, n=20) and bone scintigraphy (58%, n=19) by PRs. Magnetic resonance imaging of a symptomatic site is the imaging modality of choice for the diagnosis of CNO by (82%, n=27) of PRs. Followed by an X-ray and bone scan in (61%, n=20) and (58%, n=19) of PRs respectively. Whole-body MRI is used in diagnosis of CNO by (47%, n=15) of PRs (Figure 2). PRs reported uni-focal lesions (82%, n=27), unusual sites of presentation (79%, n=26), and multifocal lesions (30%, n=10) as the commonest reasons to perform bone biopsy in patients with suspected CNO whereas, the vertebral lesions, mandibular lesions, and clavicular lesions were least reported (about 26%) (Figure 3).

In regards to the treatment options, over half of the PRs preferred to use bisphosphonates alone in CNO patients (53%, n=17), while (43%, n=14) of PRs preferred to use non-steroidal anti-inflammatory drugs (NSAIDs) alone. Different combinations of therapy such as NSAIDs and steroids, biological therapy and bisphosphonates, disease modifying anti-rheumatic drugs (DMARDs) and bisphosphonates were preferred by (32%, n=10), (28%, n=9), (24%, n=8) of PRs respectively (Figure 5).

**Figure 4:**
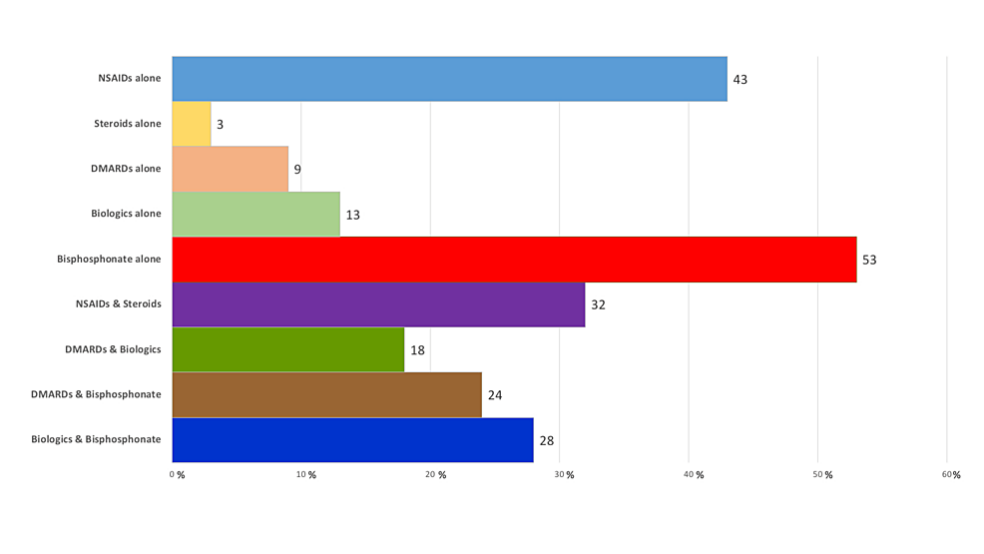
Selected medication options for the treatment of CNO.

The reasons reported for upgrading the treatment in CNO were the development of vertebral lesions (91%, n=30), the development of new MRI lesions (73%, n=24) and the elevation of the inflammatory markers (55%, n=18). Most participants selected history and physical examination (91%, n=30) to assess the disease activity in CNO, followed by estimation of the inflammatory markers by 84% (n=28), targeted symptomatic site MRI by 66% (n=22), and a whole-body MRI by 41% (n=13).

## Discussion

This is the first report, to our knowledge, that assessed the practice patterns of PRs in diagnosing and managing CNO in Saudi Arabia. We believe that we have captured variation in practice of diagnosing and treating CNO across PRs working in nine different regions across the country. The survey revealed a significant difference in disease management including clinical indications to obtain a bone biopsy, choosing the appropriate imaging modality, monitoring disease activity, and choosing the best treatment options in the view of PRs. The survey reported MRI to be the most favorable imaging modality in the diagnosis and monitoring of disease activity. Uni-focal lesions and unusual sites of presentations are the top reasons to perform bone biopsy. NSAIDs are considered the first line of CNO treatment for symptomatic relief. However, there were wide variations in practice among PRs suggesting a real need for a consensus treatment plan. The commonest three reasons for advancing a treatment in a CNO patient in our study were vertebral involvement, new lesions in MRI, and the elevation of inflammatory markers.

In our survey similar to the previous study, most of the included physicians had a reasonable duration of experience, and two third of them were often confident in diagnosing CNO [11]. Only (6%, n=3) of PRs in our previously mentioned cross sectional study saw CNO that were associated with inflammatory bowel disease (IBD), psoriasis, or uveitis even though the association between CNO and other rheumatological conditions, such as enthesitis-related arthritis, psoriasis, and IBD have been described in previous case series in paediatrics [3,13]. We speculate that the majority of these patients with other autoimmune disorders, in our survey, could possibly present to gastroenterologists or dermatologists rather than the PRs. Although the histological assessment of CNO is non-specific and invasive, bone biopsy can be helpful to rule out other differential diagnoses, such as infectious osteomyelitis, Langerhans cell histiocytosis X, and other unifocal benign or malignant primary bone tumors [10,14]. In our study, the top two reasons for considering a bone biopsy were unifocal lesions (82%) and unusual sites of presentation (79%) consistent with other reported literature on this pathology [1].

There is adequate evidence in the literature that MRI is the most important and favorable imaging modality for initial diagnosis and for follow up of CNO over the past few years. This is due to the high sensitivity of MRI in detection of symptomatic and asymptomatic lesions as well as its safety profile with regard to radiation exposure [6,7,11]. Similarly, MRI was the most commonly suggested imaging modality when CNO was suspected in our study. A whole-body MRI was suggested as the most trusted imaging modality for the diagnosis of CNO as it can reveal the diagnosis of asymptomatic lesions.

The Childhood Arthritis and Rheumatology Research Alliance (CARRA) has recently developed three consensus treatment plans (CTPs) for CNO refractory to NSAIDs and/or that with spinal lesions. Plan A includes using DMARDs (methotrexate {MTX} or sulfasalazine) while plan B includes using antitumor necrosis factor-alpha with/without MTX. Bisphosphonates represent the third CTP (plan C) [12]. PRs considered NSAIDs as the first line of treatment of CNO for symptomatic relief, although the treatment was not promising in terms of achieving complete remission of the disease [15,16]. In the Eurofever study, only a third of the patients showed a complete response to steroids [17]. In our study, only a few of the PRs preferred to use steroids as monotherapy, and many of the others preferred to use steroids in combination with NSAIDs. However, short pulses of oral glucocorticoids are considered an effective option for the management of a disease flare. Low-dose steroids are also used as bridging therapy until another effective treatment is achieved [18].

In previous reports, many have considered bisphosphonates as an effective and favorable choice for the treatment, especially for achieving complete remission [16,19,20]. Likewise, over half of the respondents in our study considered bisphosphonates alone as a treatment option for CNO. In addition to their gastrointestinal intolerance, DMARDs were found to have a low remission rate in CNO patients [15]. Only a few of our respondents suggested the use of DMARDs alone for treating CNO; and they preferred using them in combination with either bisphosphonates or biologics. Nevertheless, DMARD such as sulfasalazine can be useful in cases with IBD and enthesitis-related arthritis [3,19]. Moreover, tumor necrosis factor-alpha inhibitors were considered the most common biologic DMARDs for CNO [12]. It has been reported as a successful option for the treatment of NSAID-resistant CNO, especially if associated with other extra-osseous features, such as IBD, arthritis, and psoriasis [21].

The interleukin-1 (IL-1) receptor antagonist, anakinra, has been reported to be an effective drug for Majeed syndrome, a multi-system inflammatory disease that presents with chronic multifocal osteomyelitis, congenital dyserythropoietic anemia, with or without a neutrophilic dermatitis and deficiency of interleukin-1 receptor antagonist (DIRA) [22,23]. However, data on its use in sporadic CNO are limited, Pardeo et al. reported a cohort of nine patients who were non-responsive to NSAIDs and bisphosphonates. Of these patients, five showed a favorable response to anakinra [24]. In our study, only a few of our PRs selected biologics alone as an option of treatment for CNO patients.

The top three reasons for advancing a treatment in a CNO patient reported in our study were vertebral involvement, new lesions in MRI, and elevation of inflammatory markers, which were consistent with the findings reported by Zhao et al. [11]. As in previous literature, clinical assessment by history and physical examination in conjunction with inflammatory markers and imaging studies, particularly magnetic resonance imaging (MRI) remained to be the best tool to monitor the disease activity and follow up of CNO patients [11,12]. A long-term follow-up imaging study conducted by Voit et al. showed that a quarter of the asymptomatic patients had a persistent radiological finding on MRI, and hence, the absence of clinical symptoms does not exclude the ongoing osseous inflammation [25]. The estimation of inflammatory markers and targeted site MRI to monitor disease activity were selected by the majority of PRs in our study. However, a whole-body MRI seemed to be less used in the follow-up periods. Also, a consensus was reached in CARRA CTPS for patients to be assessed by clinical and laboratory examinations at least every three months in the first year. For a more objective assessment of the disease activity, MRI was strongly recommended at six and 12 months after any adjustment of the treatment [12].

## Conclusions

Our study results provide valuable insights into the current practices of PRs regarding the diagnosis and treatment of chronic non-bacterial osteomyelitis in Saudi Arabia. We found that there is significant variability in the diagnostic and treatment approaches used by PRs, highlighting a need for standardized guidelines to be developed in this field. By identifying the gaps in knowledge and practice, we hope that our paper will contribute to the development of consensus guidelines for the diagnosis and treatment of CNO. Such a consensus treatment plan would provide a clear framework for clinicians to manage this challenging condition, leading to improved patient outcomes and reduced healthcare costs.
